# Non-covalent Molecular Wires of Double Thiahelicene on Cu(111): A nc-AFM Study at Room Temperature

**DOI:** 10.1021/acs.jpcc.4c07662

**Published:** 2025-03-05

**Authors:** Gema Navarro-Marín, Yunbin Hu, Antoine Hinaut, Long Zhou, Shuyu Huang, Thilo Glatzel, Akimitsu Narita, Ernst Meyer

**Affiliations:** † Department of Physics, University of Basel, Klingelbergstrasse 82, 4056 Basel, Switzerland; ‡ College of Chemistry and Chemical Engineering, Central South University, Changsha 410083, China; ¶ Max Plank Institute for Polymer Research, Ackermannweg 10, 55128 Mainz, Germany; § Okinawa Institute of Science and Technology Graduate University, Okinawa 904-0495, Japan

## Abstract

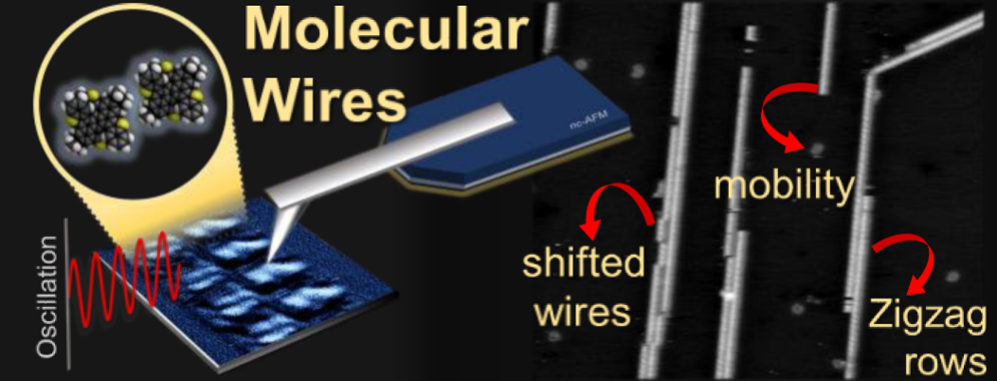

The fine control of molecules or atoms in self-assemblies on surfaces is a great challenge for future nanodevices, specially for unidimensional structure formations. In this context, our study explores the adsorption behavior of a benzo-fused double [7]thiahelicene (DT7H) on Cu(111). Using non-contact atomic force microscopy (nc-AFM) at room temperature, we prove their capability in the construction of linear-like shape adlayers. After a gentle annealing of the DT7H-copper interface, the molecules are prone to form non-covalent molecular wires which orientations are influenced by the surface symmetry. Analysis of the coverage-dependence reveals a preference for double wires at lower and intermediate densities. However, this coupling is not strong enough to prevent structural changes caused by surface mobility. Wire enlargements were induced by a further increase in surface coverage, reaching the assembly of 17 parallel molecular wires near full monolayer conditions. Finally, the electronic properties of the interface were characterized by means of Kelvin probe force microscopy (KPFM). The surface potential variations indicate a reduction of the surface work function on the regions covered by molecules, showing the functionality of this interface for optoelectronic applications.

## Introduction

In the last two decades, the speedy advance of the pioneering industry of nanoelectronics has resided in the design and construction of novel platforms capable of mimicking classical electronic functions at the nanoscale.^1,2^ In particular, researches have shown special interest in the study of molecular wires. Considerable attention has been paid to the development of novel synthetic routes and the studies of their electronic transport properties.^3^ All this investigation pursues the development of robust molecular wires, which operate as electronic pathway and interconnect the different components in a nanodevice. The analysis and construction of functional molecule–substrate interfaces take advantage of the nanotools and wide range of experimental techniques available in the surface science field. In particular, for the studies of molecular wires, mainly local probe microscopes are employed. The literature mainly discusses covalent molecular wires obtained via on-surface chemistry reactions, such as the dehalogenative and dehydrogenative routes.^4^ To a lesser extent, the formation of non-covalent molecular wires has been addressed.^5−7^ In those particular instances, the prevalence of interactions such as π–π,^8^ dipolar,^9^ and coordination bonds^10^ are responsible for the stabilization of the wire structure.

Helicene derivatives constitute promising candidates in the discipline of optoelectronics, with potential applications in fields such as optical information storage,^11^ biological probes,^12^ and information displays.^13^ Their helical structure, distorted geometry, and π-conjugated system give them intrinsic chiroptical properties, for instance, circular dichroism (CD)^14^ and circularly polarized luminescence (CPL).^15,16^ Recently, special emphasis on the study of helicene on surfaces has emerged from the opportunities to explore the phenomenon of chirality in 2D systems. By far, carbohelicene structures, specifically heptahelicene molecules on metal substrates, have been the most investigated interfaces.^17^ Nevertheless, helicene derivatives containing polar functional groups (like ciano,^18−20^ thiophene,^21−24^ and furan^25,26^) have been less analyzed. In a majority, molecules containing single helicene units have been explored, and so far only complex structures such as double helicene^27,28^ and macrocycles^29^ can be found in the literature. In contrast, a broad range of different substrates has been selected, with the aim to analyze the influence of molecule–surface interactions in molecular assemblies. The materials used comprise metal oxides,^30^ insulating^20,31^ and semiconductor surfaces to transition metals such as nickel^32^ and copper.^33^ In particular, studies on high index faces of noble metal surfaces are the most abundant.

Experimentally, the characterization of helicene molecular wires on surfaces has been analyzed by means of scanning probe microscopes. Mainly, using the scanning tunneling microscopy (STM) technique, although fewer examples using frequency-modulated non-contact AFM have been conducted.^8,20^ Submolecular resolution and stereochemical recognition of helicene units have been achieved using low-temperature STM. Experimental conditions in which molecular diffusion is reduced and 2D crystallization of the molecules is usually observed for a coverage close to 1 ML.^17^ In particular, for the deposit of racemic mixtures, the formation of zigzag rows of alternated enantiomer pairs has usually been observed. On the other hand, a search in the literature revealed only few studies carried out at room temperature.^20,21,24,29,31,32^ As a result of the high thermal mobility, visualization of helicene in this regime is quite challenging. It is likely to see them trapped at step edges or forming a 2D gas phase. Therefore, the cooling of the sample has generally been necessary to promote molecular aggregation. Among the few experiments performed at room temperature, molecular wire formation has been seen mostly for molecules containing polar groups.^5,7,8,20,21,24^ However, it is not yet clear how the number and spatial distribution of functional groups in a helicene backbone influence the self-assembled pattern.

**Figure 1:**
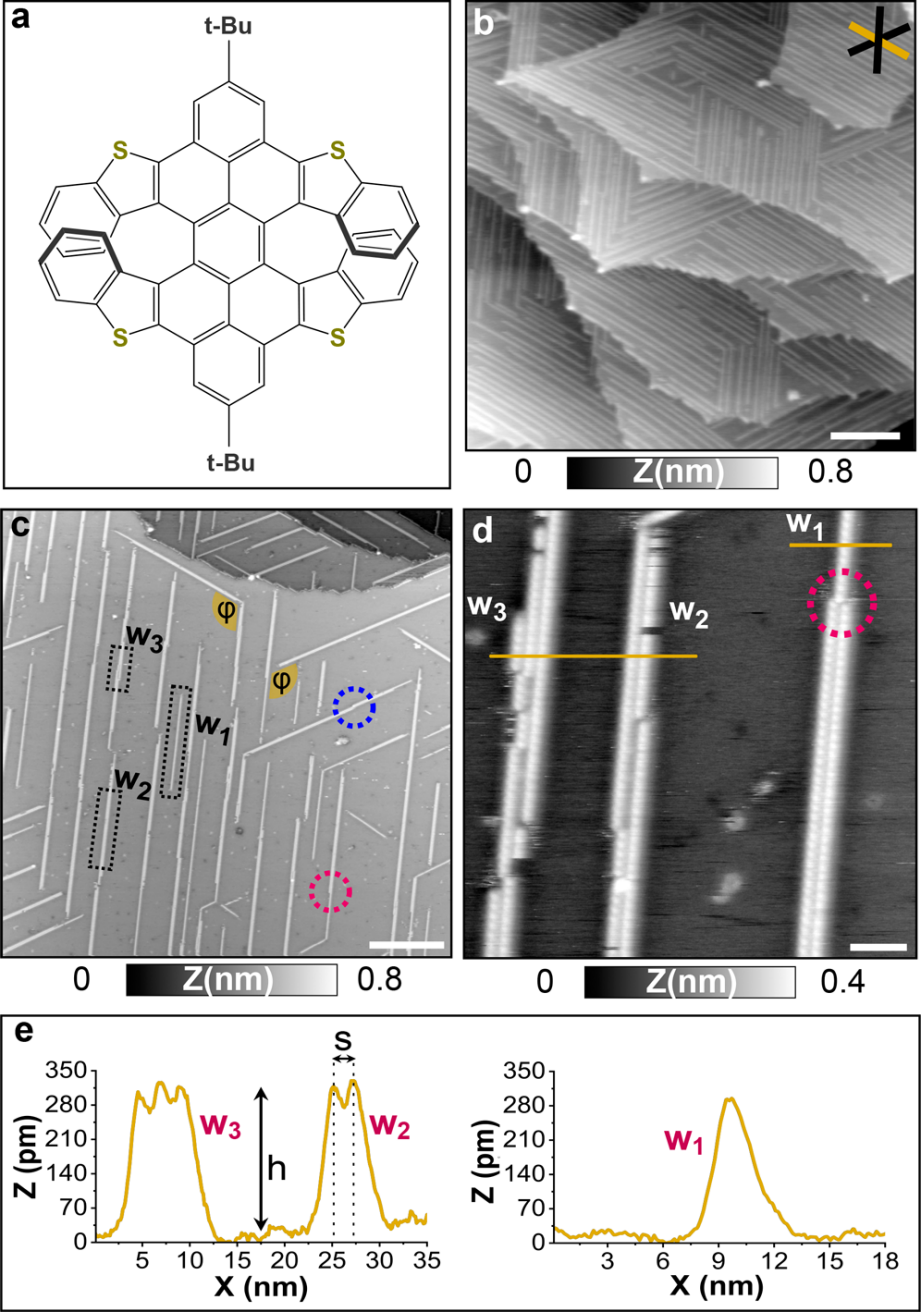
DT7H molecules and wire formation on Cu(111). (a) Schematic representation of the molecular structure of benzo-fused double [7]thiahelicene (DT7H). The molecules contain four electron-rich heterocycles (thiophene rings) and two bulky *t*-Bu groups on the extreme sides. (b) Large-scale topography of the Cu(111) terraces decorated with DT7H nanowires, measured by room temperature nc-AFM. (c) Zoomed-in image on a terrace; wires with different widths (dashed rectangles) and structural changes (dashed circles) can be observed. (d) Detailed view of the triple, double, and single molecular wires. (e) Line profiles on triple, double, and single wire sections. Average height (h) and separation distance between adjacent wires (s). Scale bars: (b) 150 nm, (c) 100 nm, and (d) 10 nm. Measurement parameters: (b) *A*_1_ = 3 nm, Δ*f*_1_ = −30 Hz, *f*_1_ = 164,293 Hz, (c,d) *A*_2_ = 800 pm, Δ*f*_2_ = −7 Hz, *f*_2_ = 1,017,360 Hz.

In the present work, we investigate the adsorption of a racemic mixture of double thiahelicene on Cu(111). The chemical structure of the studied benzo-fused double thia[7]helicene (DT7H) is illustrated in Figure 1a. The DT7H molecules absorb on Cu(111) at room temperature, forming non-covalent molecular wires. The molecules keep this linear-shaped configuration despite a further increase in the coverage, reaching maximum multiplicity at densities close to the monolayer. In our study, the dynamic and width-coverage dependence of the molecular wires were characterized by means of nc-AFM. With the aid of the multipass technique, molecular resolution was achieved, showing the formation of zigzag molecular chains. Finally, we report the formation of a surface dipole moment based on the negatively shifted contact potential difference (CPD) observed in Kelvin probe force microscopy (KPFM) measurements.

## Materials and Methods

The experiments and sample preparation were carried out in an ultrahigh vacuum (UHV) system operated at room temperature. The setup consists of a home-built nc-AFM, controlled by a Nanonis RC4.5 interface. Pretreated silicon NCLV probes from Bruker with nominal *f*_1_ = 190 kHz and *k* = 48 N/m were used. During the measurements, the cantilevers were excited simultaneously at the first or second eigenmode, along with the first torsional flexural mode.

KPFM images were conducted using the force gradient-sensitive frequency modulation method (FM), specifically FM sideband detection. In this method, an electrical excitation signal (external alternating voltage *V*_ac_ along with a dc voltage *V*_dc_) is applied between the tip and the sample. This perturbation generates an oscillating electric field that modulates the long-range electrostatic force and its gradient, leading to the modulation of the mechanical oscillation of the cantilever. In the frequency domain, the modulation is reflected in the appearance of side bands around the mechanical oscillation of the cantilever. The amplitude of the sideband at the *ac*-frequency (ω_ac_) is directly proportional to a factor (*V*_dc_ – *V*_CPD_), this amplitude can be tracked with the aid of a Lock-in amplifier. In this method, the nullification of the side bands by the application of the adequate *dc* bias (*V*_dc_ = *V*_CPD_) allows us to determine the CPD value. Our FM-KPFM images were acquired with the digital lock-in module incorporated in the Nanonis controller. The modulation frequency ω_ac_ was selected below the value of the first mechanical eigenmode (off-resonance modulation) to avoid crosstalk with the topography signal. We used an experimental configuration where the tip was grounded and the dc voltage was applied to the sample.

The monocrystalline Cu(111) substrate purchased from Mateck was cleaned by repeated cycles of Ar^+^ sputtering and annealing to 873 K. A racemic mixture of DT7H molecules was sublimated from a standard Knudsen cell evaporator at 573 K and deposited onto the clean surface, held at room temperature. Afterward, the interface was annealed at 373 K to promote faster formation of the wires. This whole procedure was performed in a preparation chamber at a base pressure below 10^–10^ mbar. The synthesis and characterization of the DT7H compounds have been done following a methodology previously reported.^34^

## Results and Discussion

Non-covalent molecular wires are visible on the Cu(111) surface after the deposition of the DT7H molecules at room temperature. As can be seen in the large-scale overview image in Figure 1b, the linear-shaped motifs are homogeneously spread on the terraces, covering 7% of the surface area. In particular, for the local region depicted in Figure 1b, the majority of the wires (58%) were observed aligned with the orientation highlighted with golden color. It is worth mentioning that this surface topography was obtained after a gentle annealing of 80 °C. Immediately after deposition, molecular wires were visible; however, a larger amount and elongated wires were observed after the annealing treatment (see Figure S1). Such oriented wire formation necessarily involves an influence of the Cu(111) substrate symmetry but is also related to the adsorption strength since we have not observed such configuration after deposition on Ag(111) (see Figure S2).

The notable increment in the number of wires after annealing suggests a thermally activated process. However, the possibility of surface-assisted reactions and/or the formation of coordination bonds should also be taken into account during the analysis. Copper surfaces are well-known to be highly reactive toward sulfur-containing species. In particular, for thiophene units, ring openings have been reported at low activation temperatures such as 150 °C.^35^ Likewise, the large number of copper adatoms available at room temperature makes the surface suitable for the formation of organometallic structures. In our study, both options were set aside based on the low temperature used and the high surface mobility retained by DT7H molecules. During the scanning, a continuous attachment and detachment of molecules from the wire frameworks was observed, which is not consistent with covalent or coordinated bounded linear structures.

The molecular wires are measured up to 500 nm in length with an average value of around 190 ± 89 nm. The wires are observed to be interrupted by the surface step-edges and other wires, which have an influence on the measured average length (see Figure S3 for discussion). This unidimensional growth differs from previous studies of carbohelicene derivatives on metal surfaces, where no long-range ordered structures have been reported at room temperature due to the high mobility. Nevertheless, for sulfur-containing helicene molecules, bidimensional adlayers have been observed.^21,36,37^ In our case, the DT7H wire formation illustrates the promotion of intermolecular interactions due to the inclusion of the thiophene ring. However, the lack of lateral growth and wire extension along the surface directions indicates the predominant influence of the substrate.

Further information about the self-assembly can be obtained from a close-up scan on a terrace (Figure 1c). Partially covered step edges can be seen; however, those sites do not constitute either nucleation points or initial traps for the growth of the wires (see Figure S1d). In addition, small mobile motives are distinguished among the molecular chains. The lengths of these dot-shaped features range between two and three molecular sizes. In general, they were visualized nearby surface defects (pits) and seemed to be anchored to them. Wire orientation and morphology are also easily visible in Figure 1c. An angle φ of 120° ± 2° between the wire growth directions was determined. The 3-fold symmetry of the wires reflects the symmetry of the substrate and confirms that molecule–substrate interactions prevail over the intermolecular ones. Looking in more detail at the wire morphologies, we can distinguish wire sections of different widths (pointed out with *w*_1_, *w*_2,_ and *w*_3_ black dashed rectangular shapes). The notation used is in agreement with the increasing width of the wires. Furthermore, some dislocations and defects are also visible in the structure of the wires. For instance, one dislocation has been highlighted with a blue dashed circle, where the shifting of the upper section can be seen. Moreover, usually in the longest chains, a width change along the same wire occurs (see the purple dashed circle).

A close-up of parallel molecular wires is depicted in Figure 1d. From left to right, sections of triple (*w*_3_), double (*w*_2_), and single (*w*_1_) wires are shown. Line profiles on the wires are illustrated in Figure 1e, indicating an average height (*h*) of 310 pm. Specifically, for the multicolumn wires (*w*_2_ and *w*_3_), a separation distance (*s*) of 2.2 ± 0.1 nm is measured. In addition, structural defects and dislocations are visible in more detail. For example, along the double wire (middle part of the image), molecular vacancies and misalignment between adjacent molecular rows are seen. Wire width change is also observed and emphasized with a purple dashed circle, indicating switching from a single to a double wire. Moreover, fuzzy lines are also distinguished, pointing to the instability of some molecules. To note, the double wire formation is predominant on the surface, whereas triple formation is scarce.

**Figure 2:**
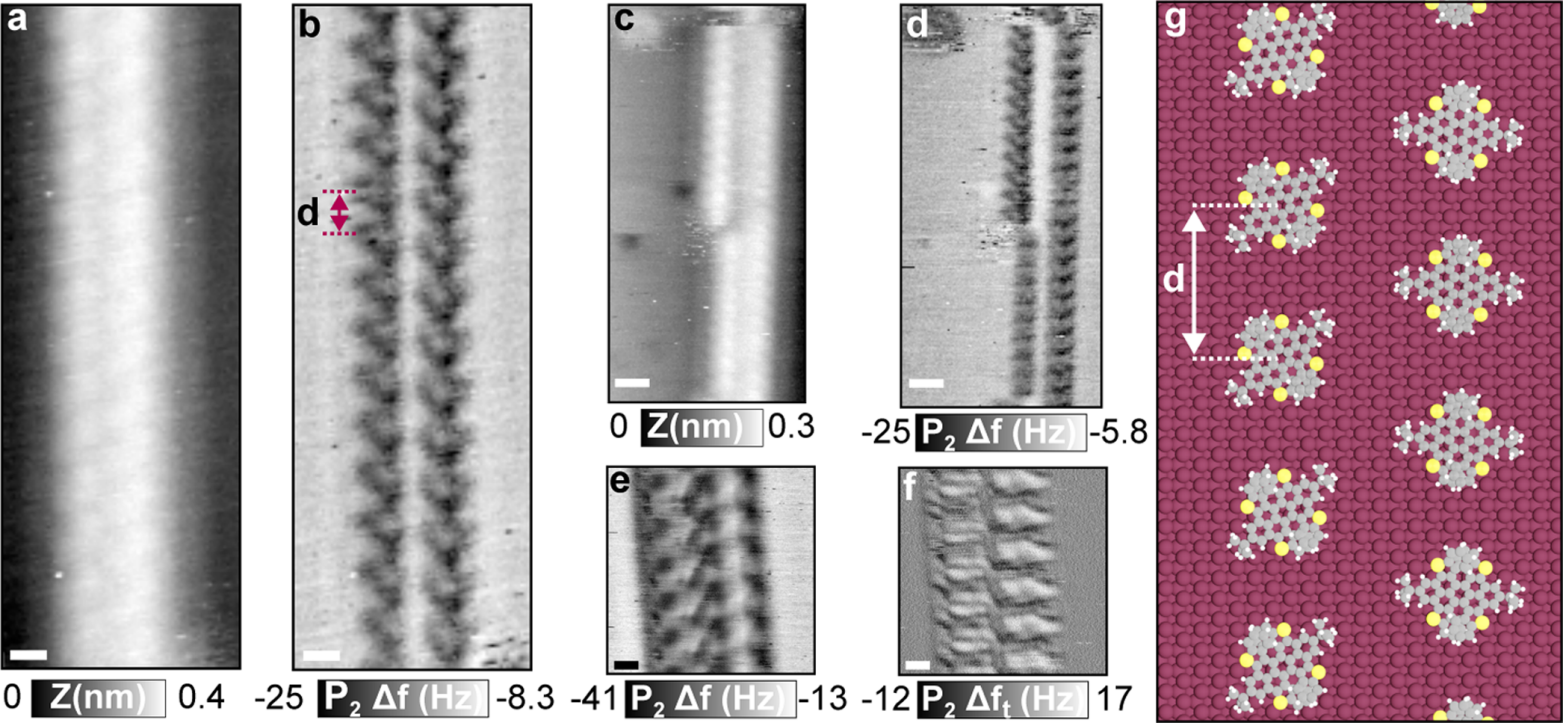
High-resolution nc-AFM image of molecular wires using the multipass-AFM method. (a,b) Topographic and frequency shift images of a defect-free double wire acquired in the first and second scanning paths, respectively. (c,d) Detail view of a shifted molecular wire section. (e,f) Molecular resolution images of the wires using the frequency shift and torsional channels measured during the second scanning path. (g) Schematic representation of the molecules in a double wire with two possible molecular orientations. Scale bars: (a,b) 2 nm, (c,d) 2.5 nm, and (e,f) 1 nm. Measurement parameters: *A*_2_ = 800 pm, *f*_2_ = 1,017,360 Hz, and *A*_*t*_ = 80 pm, *f*_*t*_ = 1,574,747 Hz, (a,b) Δ*f*_2_ = −5 Hz and *z*_offset_ = −180 pm, (c,d) Δ*f*_2_ = −5 Hz and *z*_offset_ = −150 pm, (e,f) Δ*f*_2_ = −7 Hz and *z*_offset_ = −200 pm.

Insights of the wire structures are shown in Figure 2. The individual molecules forming a double wire are visible as a bright protrusion in the topography image of Figure 2a. Better resolution is achieved with the corresponding second pass frequency shift image^38−41^ displayed in Figure 2b. In it, all the molecules along each row present the same contrast, indicating identical adsorption geometry and a defect-free part of the wire. Similar ordered structures have been observed upon the adsorption of a racemic mixture of helicene molecules on other (111)-oriented metal substrates.^17,22,23^ From the frequency shift image, a periodicity of *d* = 1.9 nm of DT7H molecules along the rows was determined. Furthermore, the negative frequency shift values and dark contrast observed on top of the molecules indicate a less attractive tip–sample interaction on the wires.^42^

AFM images of a defective double wire were collected using the same technique (see Figure 2c,d). On the middle top section of the image, a lateral shift between adjacent rows is seen, being more noticeable in the frequency shift image. The group formed by eleven DT7H molecules is located at a distance of 3 nm far from the main structure of the double wire. Furthermore, the shifted molecules seem to be on top of different adsorption sites because the zigzag configuration is not observed in this section. In addition, some vacancies and mobile molecules are also visible.

Molecular resolution was achieved after a further approach of the tip (*z*_offset_ = −200 pm). Frequency and torsional high-resolution images of a double wire region are illustrated in Figure 2e,f. Based on these images, a schematic is proposed for the molecular arrangement in the double wire, in which two possible adsorption configurations have been drawn (Figure 2g). At first sight, the torsional image presented in Figure 2f suggests that the molecules along the row present the same chirality, and double wires composed of enantiomer pairs could be considered. However, even though multiple examples of homochiral chain formation are available in the literature, this assumption should be analyzed with caution. In our particular case, the stereochemical or absolute handedness recognition of the molecules is hindered by the limited resolution under our experimental conditions. Therefore, the formation of homochiral or heterochiral double wires cannot be discriminated. Moreover, different molecular adsorption geometries should also be considered as another possible explanation for this result. This assumption is illustrated in the schematic in Figure 2g, based on the frequency shift image acquired in image 2e.

Summarizing, based on the measured height profile and molecular arrangement in the wires, we can infer a flat adsorption of the DT7H molecules. This adsorption geometry enhances the molecule–surface interaction and enables interaction between adjacent molecules. These intermolecular interactions are evidenced by the long extension of the wires and lateral growth by multicolumn wire formation. The combination and prevalence of such non-covalent interactions are responsible for the 3-fold symmetry of the wires.

### Wire Mobility

**Figure 3:**
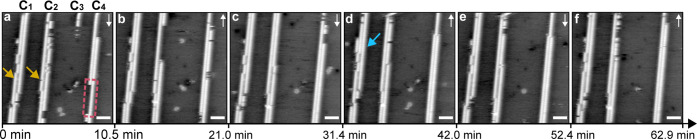
Time evolution of the molecular wires. (a–f) Sequence of topography images. Acquisition time and scanning directions are displayed in the schematic on the bottom and inset of each figure. Yellow and blue arrows point to shifted sections and triple wire formation, respectively. Scale bar: 10 nm. Measurement parameters: *A*_2_ = 800 pm, Δ*f*_2_ = −8 Hz, and *f*_2_ = 1,017,360 Hz. The *Z* scale of the images is 0.4 nm, going from black to white.

To analyze the evolution and stability of the different wire configurations, a time sequence of topography images over 3 different wires is visible in Figure 3.

Based on a closer inspection of parallel molecular wires, some conclusions can be drawn (see Figure 3). First, we can infer that single chain sections, see the purple dashed rectangle region, are transitional states for double wire formation. This is visible in the c_2_ and c_4_ double wires, where the empty gaps are constantly filled. Second, triple wire sections, such as in Figure 3d, have a shorter lifetime, which is the reason why they are rarely observed. Finally, we can also conclude that shifted sections (pointed out with yellow arrows in Figure 3a) are stable adsorption configurations for the molecules. The last statement can be confirmed by the shifted sections in the c_1_ and c_2_ columns. They remain almost intact during this close-up sequence, and in particular, for the wires in c_1_, the two shifted regions are present in a previous sequence in Figure S4 (see blue rectangle).

Noisy lines parallel to the scan direction typically observed in adsorbate surface diffusion are also visible. The total number of molecules constituting the wires is constantly changing, starting from 230 molecules (first scan), reaching a maximum of 248 (fourth scan), and finishing with a total of 238 molecules. Due to the high mobility, an independent tracking of the molecules was not possible. Moreover, taking into account the number of molecules in each column, we could not prove a direct interchange between neighboring wires. In our time frame, the addition and removal of odd and even numbers of molecules from the wire skeleton were visualized (see schematic in Figure S5). Therefore, the identification of the number of molecules that compose the diffusive unit set was not possible. Using the corresponding dissipation images (see Figure S5), we did not detect features or local increases at the wire edges, suggesting that molecular displacements are not tip-induced.^43^ Therefore, we infer that the mobile 2D gas phase is the main source of contribution to the morphological changes in the structure of the wires.

Although wire morphological structures are modified by surface diffusion, the process plays a key role in the visualization of the wires at room temperature. The diffusion assists the intermolecular interactions that lead to the formation of the wires. However, the dynamism of the system is highly limited by the surface.

### Coverage Influence

**Figure 4:**
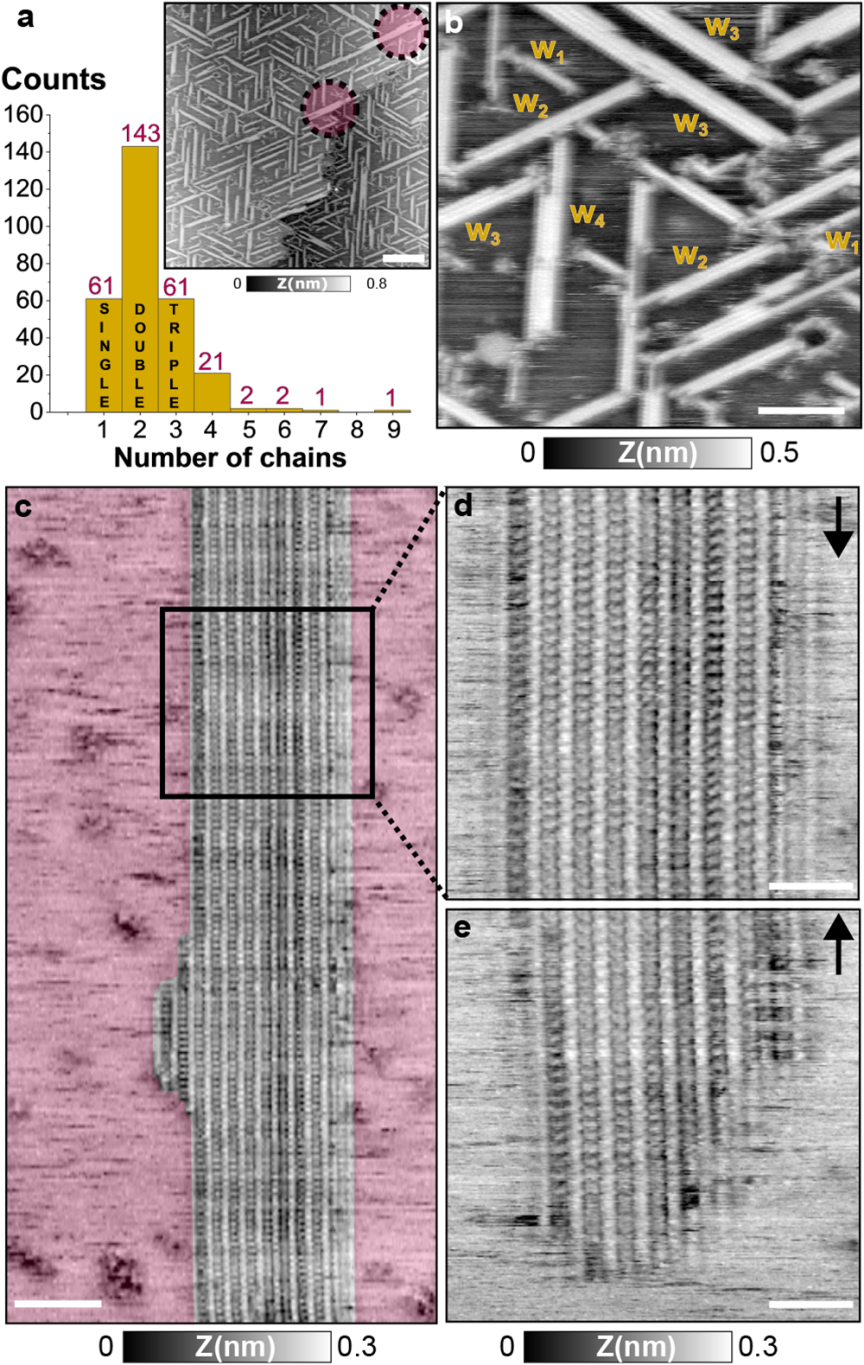
DT7H molecules at higher coverages on Cu(111). (a) Width distribution of the wires at intermediate coverage (inset nc-AFM topography image of the surface, regions with wider wires have been emphasized with dashed circles). (b) Close-up on a terrace at intermediate coverage showing the width increase and coexistence of multiple wires, the subscripts indicate the number of single molecular chains contained. (c) Molecular wire at coverage close to the monolayer. (d,e) Consecutive images acquired on the square region highlighted in (c). Scale bar: (a) 100 nm, (b) 30 nm, (c) 20 nm, and (d,e) 10 nm. Measurement parameters: (a,b) *A*_1_ = 4 nm, Δ*f*_1_ = −6 Hz, *f*_1_ = 178,684 Hz, and *V*_bias_ = 600 mV, (c–e) *A*_1_ = 5 nm, Δ*f*_1_ = −5 Hz, *f*_1_ = 185,130 Hz, and *V*_bias_ = 308 mV.

The increase in surface coverage leads to the formation of shorter wires, following the same equivalent orientations. The reduction in length observed for higher coverage can be explained by the increased probability of molecular wires encountering other neighboring wires or molecules, which would stop their elongations. The inset image in Figure 4a illustrates a copper terrace with 24% of the surface area covered by molecules. In contrast to the low coverage regime, molecular wires attached to step edges are seen. Furthermore, the number and size of molecular clusters among the wires also raised (see Figure 4a,b). Noteworthy is the formation of wider molecular wires. An increase in the multiplicity, the number of chains per wire, is measured and emphasized by the width distribution and close-up image in Figure 4a,b. The histogram displayed in Figure 4a indicates that the double-wire configuration is still by far the most predominant on the surface. In contrast, under these conditions, triple wires are longer and more stable; besides, their amount matches the number of single ones. To a lesser extent, a higher order of multiplicity is observed, reaching the formation of nine non-covalent bonding single chains (see highlighted circular regions in Figure 4a). During the scanning, moderate changes were visible in areas covered with aggregates and isolated single wires, suggesting that diffusion of the molecules is still present.

Reaching higher surface coverage induces the formation of even larger and wider molecular wires, as can be seen with the nc-AFM topography image in Figure 4c. Herein, an elongated wire of 35 nm width, equivalent to 17 parallel molecular chains, is visualized. The wire separation *s* has reduced to 1.9 nm, and the periodicity *d* along the molecular chains also decreases to 1.6 nm (see profiles in Figure S6). The height of the wire is also reduced compared to the low-coverage regime, and a drop of 50 pm is measured on the wire with respect to the surrounding area. Mobile species seem to be covering this adjacent area (red region). The observed spikes in the scanning lines and holes are in agreement with the topography profile, implying the presence of a disordered molecular layer. Moreover, it is also noticeable that some molecules at the edges of the wire present a fade contrast, a sign of a possible switching between order (in the wire) and disordered (in the surrounding area) adsorptions. The presence of such disordered molecular phase can favor the confinement of the molecules in favor of the formation of larger molecular wires.

Despite this confinement, the molecules are still mobile, and wires can appear (disappear) or extend (shrink) over time. For instance, such evolution is illustrated in the two consecutive topography images (Figure 4d,e) acquired in the rectangular region highlighted in the previous image, showing the disappearance of the wire. This switching state of the molecules between the disordered and ordered regions indicates that the molecules can still diffuse on the surface, implying a coverage below the monolayer. Still, the observation of the spikes over the disordered area in the topography image, as well as the reduced height difference between the wire and the surrounding area (see height profile in Figure S6), suggest that many diffusing molecules are present on the terraces. These molecules would become a source for the growth of the wire. Under these conditions, the disordered DT7H layer saturates the surface, causing the yield of molecular wire formation to be deficient in comparison with previous coverage.

### KPFM Measurements

**Figure 5:**
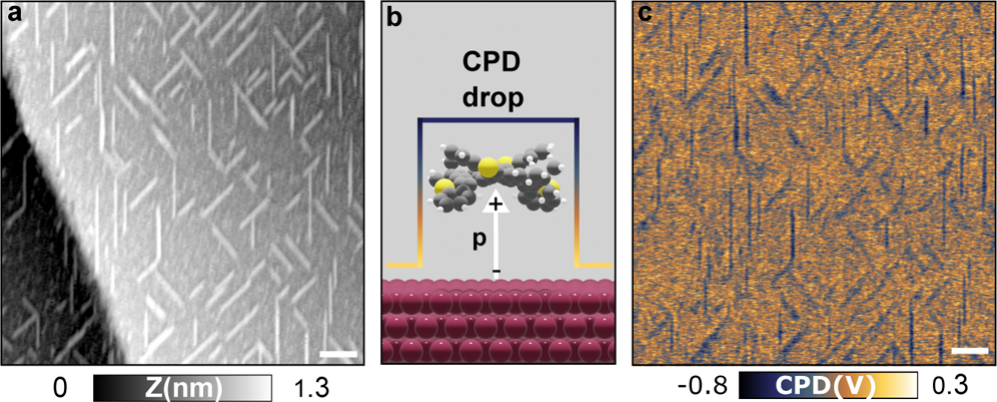
Topography (a), schematic representation (b), and FM-KPFM (c) images of DT7H molecular wires on Cu(111) at low coverage. In the 3D schematic, the offset of the molecule has been enlarged for better illustration; the sizes of the chemical elements (sulfur, carbon, and copper) are displayed in agreement with their atomic radios. Scale bar: 50 nm. Measurement parameters: *A*_1_ = 2 nm, Δ*f*_1_ = −19 Hz, *f*_1_ = 176,477 Hz, *V*_ac_ = 600 mV, and *f*_ac_ = 100 Hz.

In order to obtain information about the electronic properties of the system, FM-KPFM measurements were performed. Topography and CPD images acquired simultaneously on the wires at low coverage are shown in Figure 5. The contrast variation observed in Figure 5c indicates that the CPD and hence the surface work function decrease across the molecules, being more evident at the double and triple wire locations (see schematic representation in Figure 5b). A work function difference of −382 mV was extracted from the KPFM image. Similar behavior was also observed on the Ag(111) surface, where a CPD drop of −349 mV was measured between the molecular island and the uncovered substrate (see Figure S2).

At metal/organic interfaces, this modification of the surface work function is usually associated with the formation of a surface dipole moment. Phenomena such as chemisorption, charge transfer and rearrangement, and molecular polarization among others^44^ can be responsible for the arising of this interface dipole. Negative CPD values have been previously reported in molecular assemblies of aromatic compounds where a flat configuration has been adopted.^45,46^ Likewise, the lie-down adsorption geometry adopted by the DT7H molecules enables interaction of the extended π system with the underlying substrate. This perturbation/redistribution of the surface electron density due to the Pauli repulsion with the delocalized molecular electronic cloud is known as the “push back” effect and reduces the work function of the system. On the other hand, the electronic configuration of single molecules can be modified in response to the polarizability of the surrounding molecular layer. In particular, for physisorbed adlayers where intermolecular interactions are relevant, the charge screening by neighboring molecules has been shown to significantly impact the electronic structure (e.g., energy level alignment) of the molecule/surface system.^47^ This phenomenon may also alternatively be responsible for the CPD difference detected. The proportional relation between the CPD and the difference in dipole moment densities (Δ*p*) is given by .^5,39^ Taking into account the experimental value of Δ*V*_CPD_ on copper, a difference in dipole moment densities of −1.1 D nm^–2^ was obtained. Finally, the contrast observed in Figure 5c, allows us to predict an average dipole moment oriented from the surface toward the molecular layer (see schematic in Figure 5b).

## Conclusions

In summary, we studied the formation of non-covalent molecular wires upon adsorption of a racemic mixture of double thiahelicenes on Cu(111) at room temperature. The wires are found to be very elongated, reaching up to a 500 nm length. Their growth is limited by step edges and surface coverage, besides their alignment reflects the surface symmetry. At low and intermediate coverages, we observed that wires are preferentially formed by two individual molecular rows. Using multifrequency nc-AFM imaging, as well as the multipass method, we studied the molecular configuration in the wires. Our findings indicate that along defect-free molecular rows, the adsorption configuration is preserved, whereas it can vary when an adjacent molecular row is nearby while keeping the periodicity.

In the next step, the time evolution of the wires was analyzed. The equilibrium between the molecules diffusing on the surface and the molecules stabilized in wires was reflected in the incorporation and removal of them from the wire frameworks. Further increase in the surface coverage allowed us to show the influence of the number of surrounding diffusing molecules on the wire structure, with the growth of larger wires being favored. We attribute this enlargement to a reduction in mobility due to the confinement arising from the presence of the disordered layer.

We obtained complementary information from the mapping of the local electrostatic potential of the interface. The formation of a local dipole moment was confirmed by the arising of a negative local contact potential difference in KPFM measurements. This functionalized interface could be beneficial in applications such as field-effect transistors (FETs) and organic light-emitting diodes (OLEDs) where low-work-function electrodes are desired.

## Supporting Information Available

DT7H molecular wires on Cu(111) prior to annealing treatment; adsorption of DT7H on Ag(111): topography and KPFM images; wires mobility on Cu(111); and dissipation images of DT7H wires on Cu(111) (PDF)
