# Supplementary material for: Non-covalent Molecular Wires of Double Thiahelicene on Cu(111): A nc-AFM Study at Room Temperature
Source: J Phys Chem C Nanomater Interfaces. 2025 Mar 5;129(11):5637–44. doi: 10.1021/acs.jpcc.4c07662 (PMC11931536; doi:10.1021/acs.jpcc.4c07662)
Supplement: Supplementary file 1 — jp4c07662_si_001.pdf [file jp4c07662_si_001.pdf]

Non-covalent molecular wires of double thiahelicene on Cu(111): a nc-AFM study at room temperature

Gema Navarro-Marín,† Yunbin Hu,‡ Antoine Hinaut,*,† Long Zhou,‡ Shuyu Huang,† Thilo Glatzel,† Akimitsu Narita,¶,§ and Ernst Meyer*,†

†*Department of Physics, University of Basel, Klingelbergstrasse 82, 4056, Basel, Switzerland*

‡*College of Chemistry and Chemical Engineering, Central South University, Changsha 410083, China*

¶*Max Plank Institute for Polymer Research, Ackermannweg 10, 55128, Mainz, Germany*

§*Okinawa Institute of Science and Technology Graduate University, Okinawa 904-0495, Japan*

E-mail: antoine.hinaut@unibas.ch; ernst.meyer@unibas.ch

Contents

1. DT7H molecular wires on Cu(111) prior to annealing treatment. 3

2. Adsorption of DT7H on Ag(111) 4

3. Influence of terrace size on wire length 6

4. Molecular wires mobility 8

5. Molecular wires dissipation **9**

6. Molecular wires confinement **10**

References **10**

1. DT7H molecular wires on Cu(111) prior to annealing treatment.

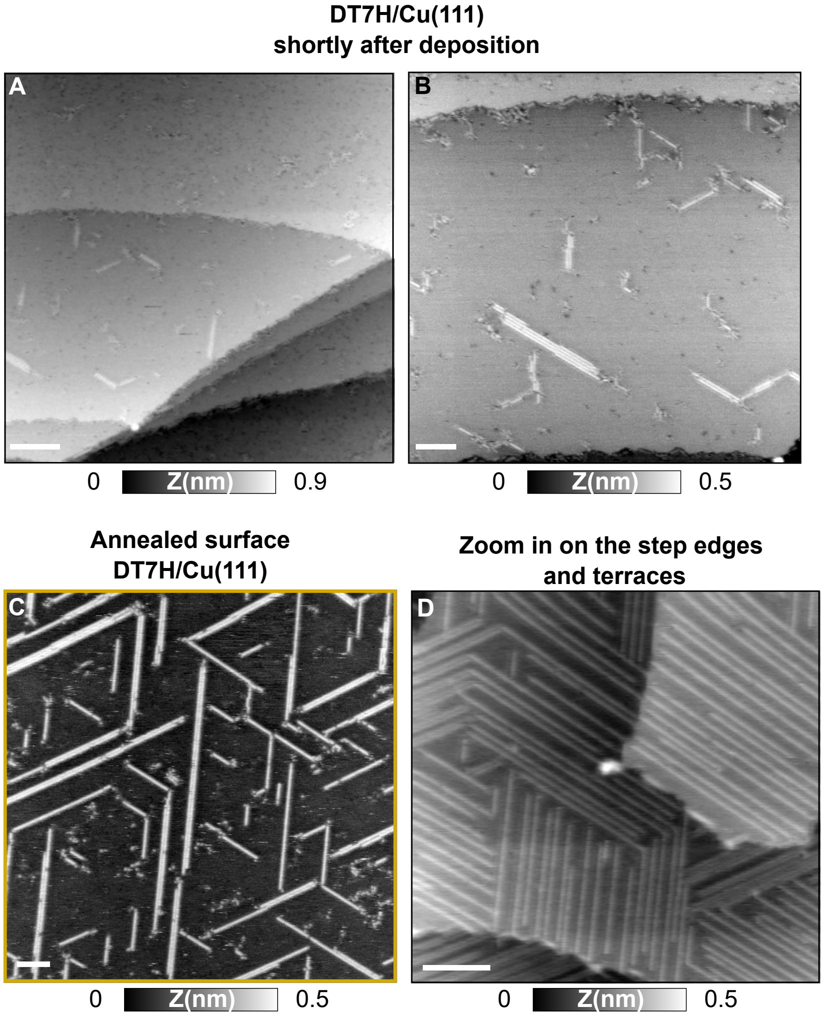

Figure S1: nc-AFM images of DT7H/Cu(111). a-b) Short and scattered DT7H molecular wires are distinguished on the copper terraces. The images were acquired immediately after the deposit of the molecules. c) Sample topography after a mild annealing (80^0C) showing the thermal activated process. An increase in the number and length of the wires is visible. d) Close-up on a terrace and step edges. Scale bar: a) 50 nm, b-c) 25nm and d) 100 nm. Measurement parameters: a-b) A= 4 nm, Δf= -14 Hz, f_1 = 180184 Hz, c) A= 5 nm, Δf= -9 Hz, f_1 = 178736 Hz and d) A= 2 nm, Δf= -25 Hz, f_1 = 164293 Hz.

2. Adsorption of DT7H on Ag(111)

In order to study the influence of the surface reactivity in the adsorption pattern, DT7H molecules were also deposited on Ag(111) substrate. In contrast to copper surface, the affinity of DT7H with silver was very low. The molecules were mainly attached to the step edges and only formation of mobile tiny islands were distinguished (see Figure S2). This outcome is in agreement with previous investigations of helicene molecules on noble metals. The weak molecule-subtract interactions enable the thermal excited molecule to surpass the energetic barrier imposed by the surface potential and being able to diffuse on it. Furthermore, a study of single thiahelicenes on Ag(111), made by means of X-ray photoelectron spectroscopy (XPS), corroborate the weakly Ag-S interaction by the presence of physisorption components in the sulphur spectra.[1] For this reason, formation of well defined DT7H overlayer is not observed. Therefore, we conclude that in the formation of DT7H molecular wires on copper, there is an influence of the substrate, perhaps related with a site-specific adsorbate-substrate interactions.

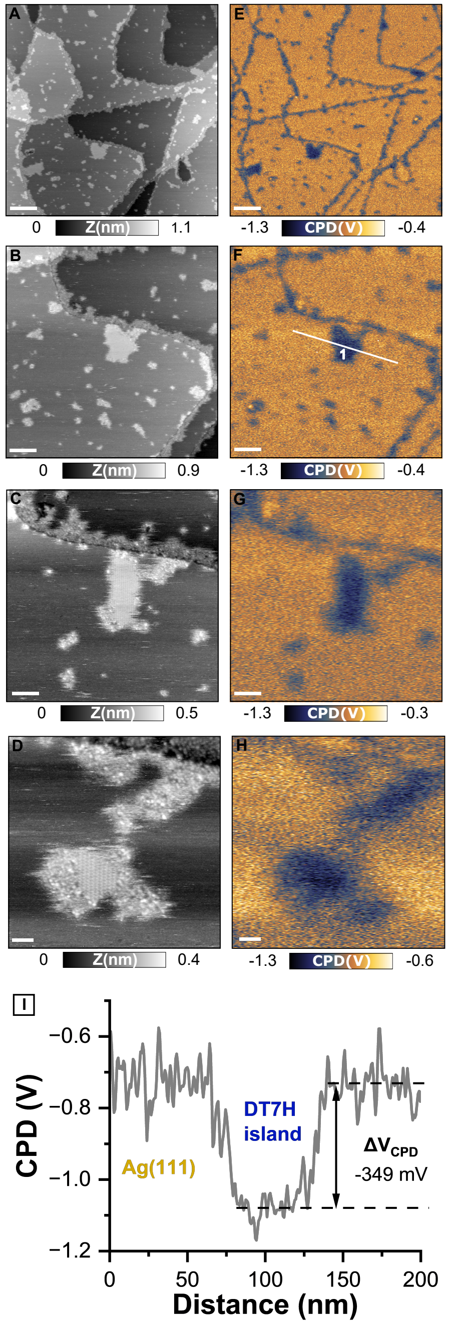

Figure S2: nc-AFM and CPD images of DT7H/Ag(111). a-d) Topography images illustrating the formation of small molecular islands and step edges fully covered. e-h) Corresponding CPD images, the molecular regions are easily distinguishable due to their strong contrast (dark blue color) and represent areas where the work function of the surface decreases. i) Line profile above a molecular island showing the CPD difference between a molecular layer and the surface. Scale bar: a) 100 nm, b) 50nm, c) 25 nm and d) 10 nm. Measurement parameters: a-b) A= 5 nm, Δf= -8 Hz, f_1 = 163834 Hz.

3. Influence of terrace size on wire length

In order to clarify the influence of the size of the terraces on the length and orientation of the wires, we have made the following analysis. In the AFM image presented in Figure S3, the complete micrograph of 1.2 μm x 1.2μm size is illustrated. By making a global analysis of the orientations (see pay chart), we can confirm that the wires mostly grow along the direction highlighted in red color (60.7 %). Similar percentages are found for the other two orientations (17.1 % and 22.3 %), with the one shown in light purple being slightly bigger. We have made a local analysis, i.e. focusing on the terraces named with the letters A,B,C,D whose extension areas are totally included in the image. As expected in these local regions, the wires following the red orientation are the most abundant per terrace ($A_{terrace}$= 22, $B_{terrace}$= 23, $C_{terrace}$= 35 and $D_{terrace}$= 63) and generally their number increases with terrace area. In particular, we can observe that their length increases considerably on narrow terraces (A terrace), but nevertheless they keep the highest average length value of 177 $\pm$ 8 nm among the four regions.

The local analysis of the two remaining orientations is in agreement with what was observed in the global study. Slightly above the number of blue wires appear the violet ones, with an abundance of 43 and 58 wires respectively, as a total sum of the regions. The analysis of the influence of the surface area of the terrace indicates that, except for terrace A, the length values of violet wires alternate around the average of 118.3 $\pm$ 7 nm. Therefore, we could confirm the dependence of the length on the terrace area for wires growing along red and violet orientations. Based on these observations, we can conclude that as the terrace area increases, the length of these wires oscillates around an average value. We also believe, that the shape of the terrace and the diffusion process present in our system has a considerable impact on the length parameter measured.

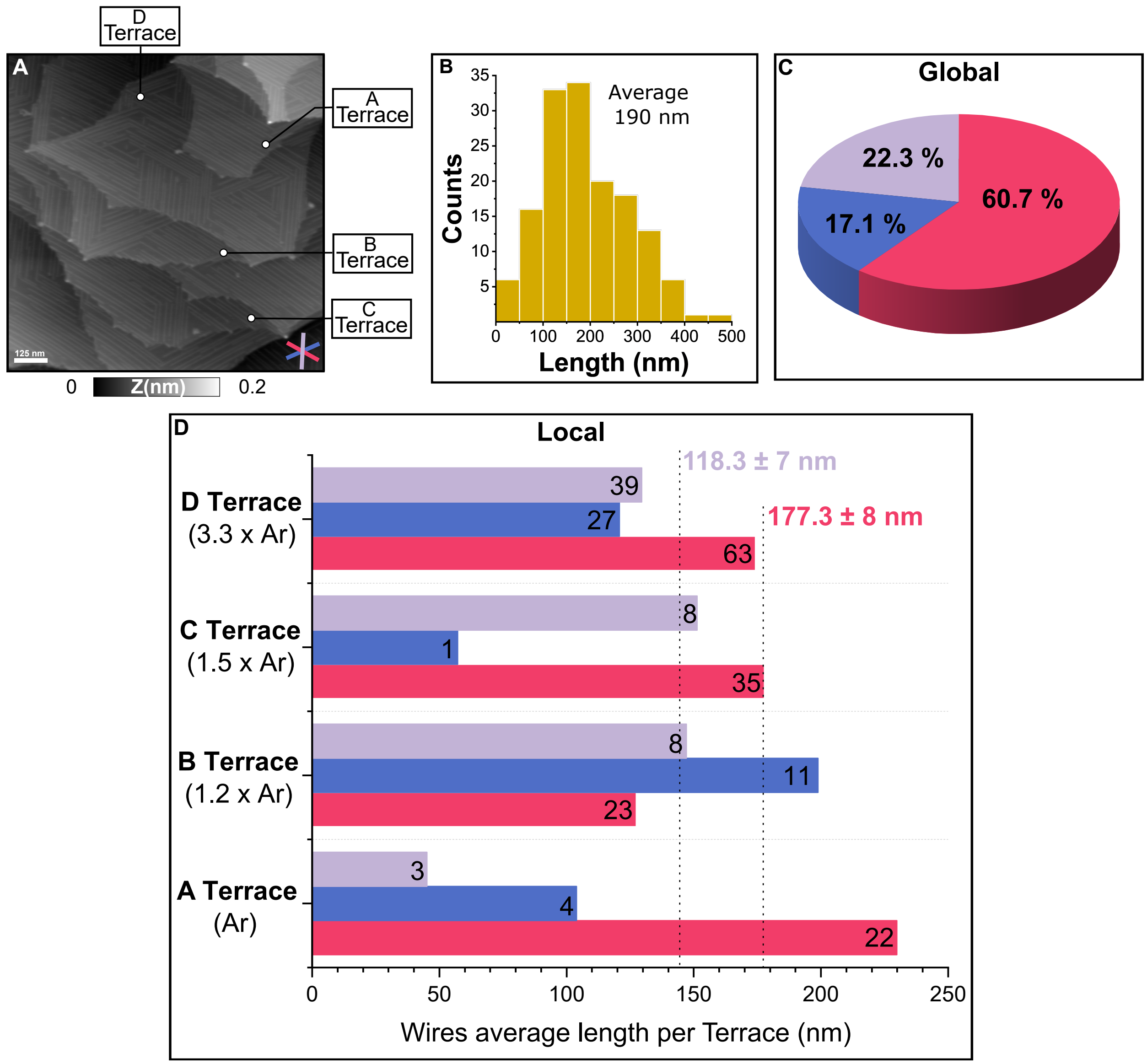

Figure S3: a) Low resolution image of 1.2 μm x 1.2 μm size, showing the wires adsorbed on Cu(111) after the annealing of the interface. Highlighted, the terraces used in the local wire extension analysis. Different orientations were emphasized with different colors (red, blue and light violet). b) Length distribution of the molecular wires. c) Pay chart illustrating the numerical proportion between the different wire orientations. d) Analysis of the impact of the area of the terraces (A, B, C, D) on the length and orientation of the wires. Area of the different terraces are reported relative to the area value of terrace A, Ar = 101771 nm^2. Inside each bar, we have placed the value corresponding to the number of wires with this orientation per terrace. Measurements parameters: a) A_1 = 3 nm, Δf_1 = -30 Hz, f_1 = 164293 Hz.

4. Molecular wires mobility

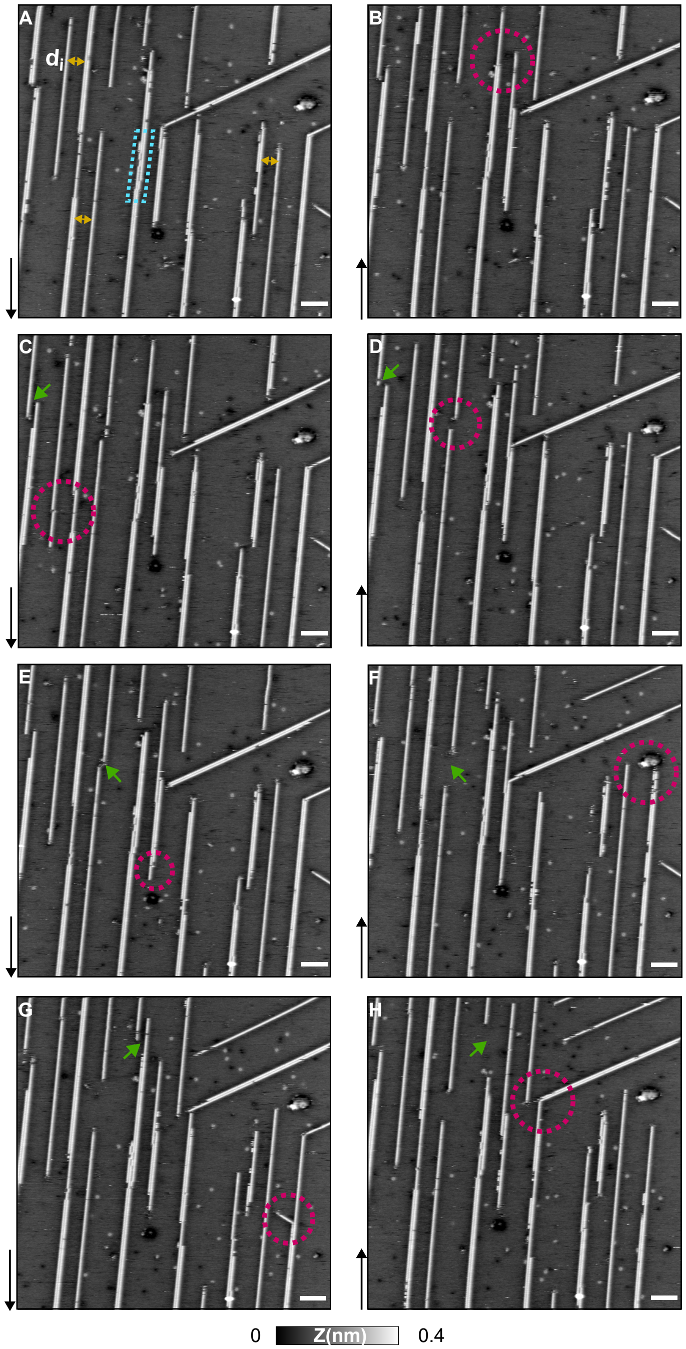

Figure S4: Time evolution of DT7H molecular wires on Cu(111). On the terrace, among the double wires, the growing of long and isolated single molecular chains is visible. Those wires can reach up 160 nm length and interactions with neighbours wires was only observed for distances shorter than $d_i \leq 17.6$ nm. Mutual repulsion among single chains sections were also observed and highlighted with green arrows. Morphological changes (attachment and detachment sections) respect to the first scan have been emphasized with purple circles. Scale bar: 25 nm. Measurements parameters: A= 800 pm, Δf= -8 Hz and f_2 = 1017360 Hz.

5. Molecular wires dissipation

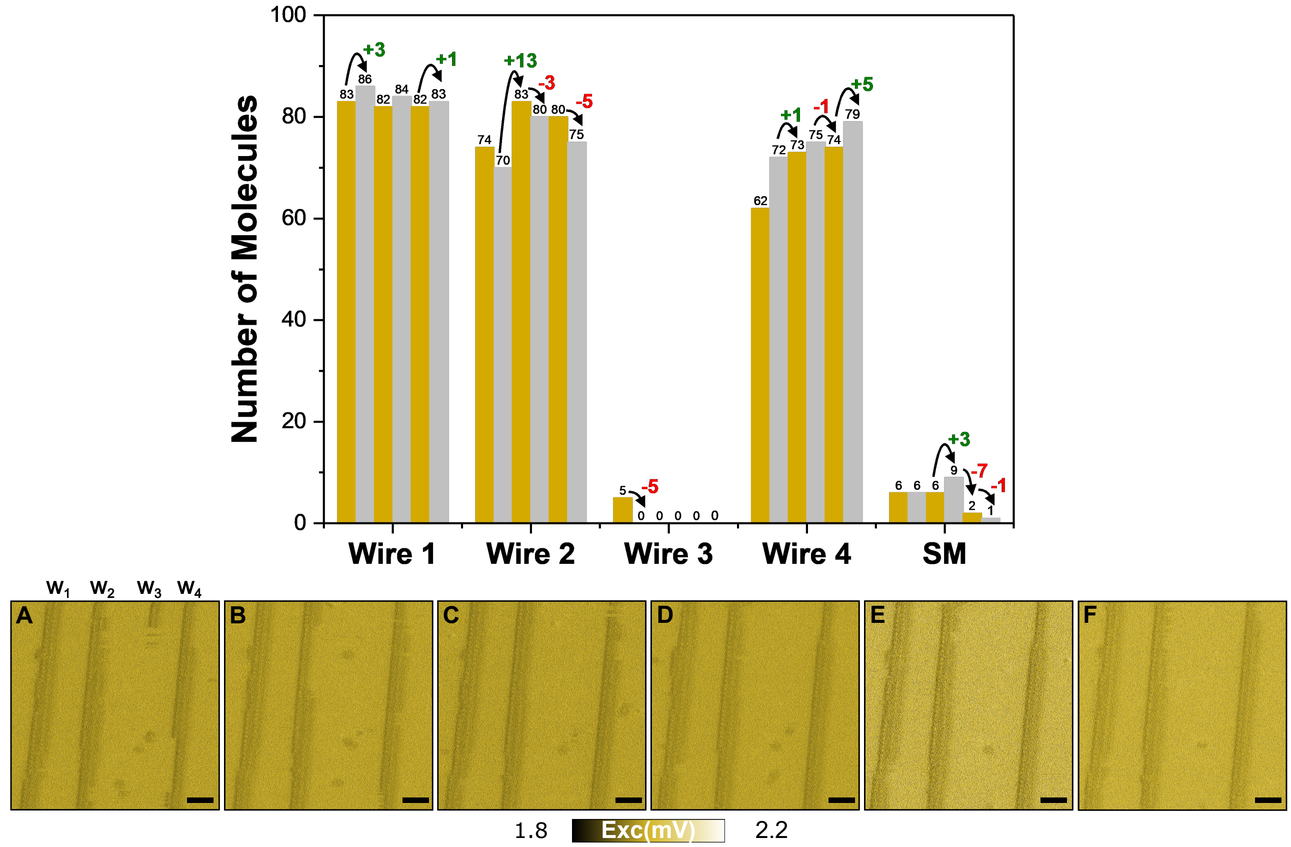

Figure S5: Schematic indicating of the composition per wire along the sequence. Addition/remotion of odd number of molecules has been highlighted with green and red color respectively. a-f) Dissipation images of parallel molecular wires adsorbed on Cu(111). The dissipated signal is lower above the molecules. No peaks or stroke lines were observed along the sequence, excluding the manipulation of the molecules by the AFM tip. Scale bar: 10 nm. Measurements parameters: A= 800 pm and Δf= -8 Hz, f_2 = 1017360 Hz.

6. Molecular wires confinement

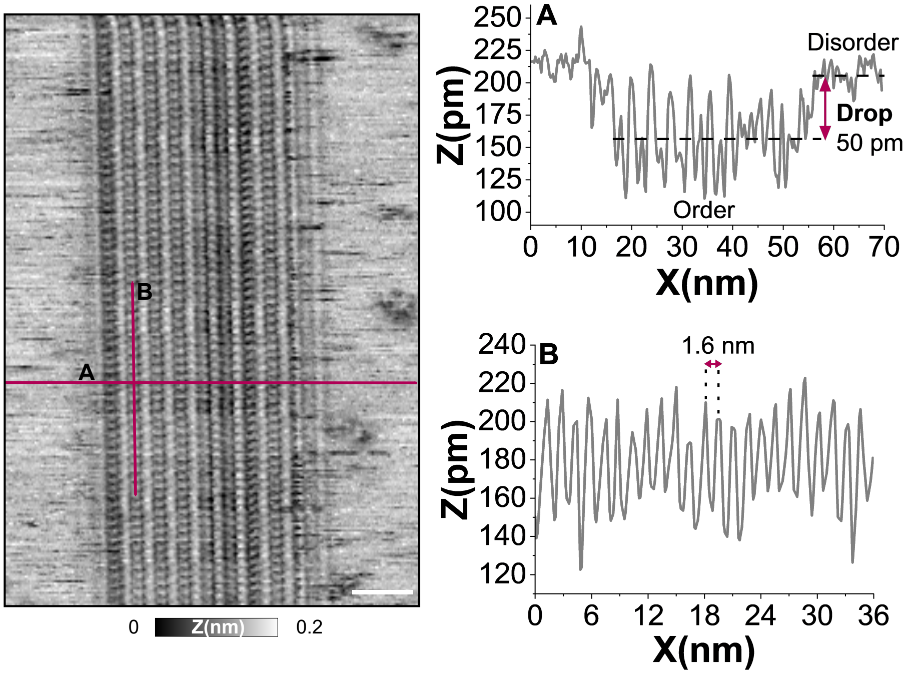

Figure S6: nc-AFM images of a multiple DT7H molecular wire on Cu(111) close to monolayer regime. Profiles extended along the directions indicated by A and B lines show a drop height of 50 pm between wires and disorder regions, besides a reduction to 1.6 nm of the periodicity. Scale bar: 10 nm. Measurements parameters: A= 5 nm and Δf= -5 Hz and f_1 = 185130 Hz.

References

(1) Krukowski, P.; Hattori, T.; Okada, M.; Piskorski, M.; Lutsyk, I.; Saito, A.; Osuga, H.; Kuwahara, Y. Study of Stereochemical Crystallization of Racemic Mixtures of [5] and [7]Thiaheterohelicene Molecules on Ag(111) Surface by Scanning Tunneling Microscopy and Raman Scattering Spectroscopy. *589*, 152860.
